# The Cultural and Contextual Adaptation Process of an Intervention to Reduce Psychological Distress in Young Adolescents Living in Lebanon

**DOI:** 10.3389/fpsyt.2020.00212

**Published:** 2020-03-23

**Authors:** Felicity L. Brown, May Aoun, Karine Taha, Frederik Steen, Pernille Hansen, Martha Bird, Katie S. Dawson, Sarah Watts, Rabih el Chammay, Marit Sijbrandij, Aiysha Malik, Mark J. D. Jordans

**Affiliations:** ^1^Research and Development Department, War Child Holland, Amsterdam, Netherlands; ^2^Amsterdam Institute of Social Science Research, University of Amsterdam, Amsterdam, Netherlands; ^3^Research and Development Department, War Child Holland, Beirut, Lebanon; ^4^The Reference Centre for Psychosocial Support, The International Federation of Red Cross and Red Crescent Societies, Copenhagen, Denmark; ^5^School of Psychology, University of New South Wales, Sydney, NSW, Australia; ^6^Independent Researcher, Geneva, Switzerland; ^7^Department of Psychiatry, Faculty of Medicine, Saint Joseph University, Beirut, Lebanon; ^8^National Mental Health Programme, Ministry of Public Health, Beirut, Lebanon; ^9^Department of Clinical, Neuro- and Developmental Psychology, World Health Organization Collaborative Center for Research and Dissemination of Psychological Interventions, Vrije Universiteit, Amsterdam, Netherlands; ^10^Oxford Institute of Clinical Psychology Training, Warneford Hospital, University of Oxford, Oxford, United Kingdom

**Keywords:** psychological intervention, cultural adaptation, low- and middle-income countries, humanitarian emergencies, armed conflict, adolescents

## Abstract

Armed conflict leads to increased risk of emotional distress among children and adolescents, and increased exposure to significant daily stressors such as poverty and community and family violence. Unfortunately, these increased risks usually occur in the context of largely unavailable mental health services. There is growing empirical support that evidence-based treatment techniques can be adapted and delivered by non-specialists with high fidelity and effectiveness. However, in order to improve feasibility, applicability, and outcomes, appropriate cultural and contextual adaptation is essential when delivering in different settings and cultures. This paper reports the adaptation process conducted on a new World Health Organization psychological intervention—Early Adolescent Skills for Emotions (EASE)—for use in the north of Lebanon. Lebanon is a middle-income country that hosts the largest number of refugees per capita globally. We conducted: i) a scoping review of literature on mental health in Lebanon, with a focus on Syrian refugees; ii) a rapid qualitative assessment with adolescents, caregivers, community members, and health professionals; iii) cognitive interviews regarding the applicability of EASE materials; iv) a psychologist review to reach optimal and consistent Arabic translation of key terms; v) “mock sessions” of the intervention with field staff and clinical psychology experts; vi) gathering feedback from the Training of Trainers workshop, and subsequent implementation of practice sessions; and vii) gathering feedback from the Training of Facilitators workshop, and subsequent implementation of practice sessions. Several changes were implemented to the materials—some were Lebanon-specific cultural adaptations, while others were incorporated into original materials as they were considered relevant for all contexts of adversity. Overall, our experience with adaptation of the EASE program in Lebanon is promising and indicates the acceptability and feasibility of a brief, non-specialist delivered intervention for adolescents and caregivers. The study informs the wider field of global mental health in terms of opportunities and challenges of adapting and implementing low-intensity psychological interventions in settings of low resources and high adversity.

## Introduction

There are over 25 million refugees worldwide, around half are children, and the majority (84%) are hosted in low- and middle-income countries (LMIC) (1). Children frequently experience significant stressors and barriers to healthy development during the refugee experience, including poverty, education interruptions, exposure to traumatic events, increased family and community violence, and child protection risks (2). These experiences contribute to a greatly increased risk for poor mental health (3). Host communities in LMICs also face similar stressors, associated with living in adversity, and these stressors impact significantly on child and adolescent mental health (3).

Alongside increased mental health needs in LMICs, a vast treatment gap exists, whereby the majority of individuals needing mental health treatment do not receive minimally adequate care (4, 5). Significant barriers to providing necessary support for children and adolescents in these settings include limited financial resources (4, 6, 7) and under-resourced professional mental health workforces. In low-income countries there are less than 2 mental health professionals per 100,000 population, compared with over 70 in high-income settings (8).

Accordingly, a large body of work has considered scalable psychological interventions to overcome the barriers to providing quality mental health care in LMICs. These interventions often utilize “task-shifting”, whereby non-specialists (people without professional clinical qualifications in mental health e.g. lay counselors, nurses, primary care providers, peers) are trained and supervised to deliver services that have historically been delivered by clinical professionals (9, 10). Furthermore, they may consist of briefer and simplified versions of existing evidence-based treatments, and reduce costs *via* alternative formats such as group-based implementation. Rather than developing disorder-specific intervention packages, focus has shifted to developing interventions that target underlying common symptoms and processes, and that are safe and effective for individuals with a range of disorders or sub-threshold distress. There is growing empirical support that these scalable low-intensity interventions can be implemented effectively by non-specialists in LMICs (11–13). The World Health Organization (WHO) is developing and testing the effectiveness of scalable psychological interventions that can be implemented by trained and supervised non-specialists in multiple cultural contexts (10). For example, Problem Management Plus, has shown efficacy in treating psychological symptoms and distress in adults, when delivered one-to-one, and in group format (14–16).

While these findings show promise for the feasibility of delivering evidence-based approaches in varying cultural settings, appropriate consideration of cultural factors is essential (17). There is evidence that culturally adapted treatments are more effective than non-adapted treatments (18–20). Although one recent meta-analysis of psychological interventions for depression in LMICs found no significant difference between adapted and non-adapted therapies (21), another meta-analysis of minimally guided treatments for depression found that treatment effects increased with additional cultural adaptations made (20). A barrier to adequately evaluating the empirical support for the process of cultural adaptation, is that the majority trials of psychological interventions in LMICs do not comprehensively outline adaptations made (22). Cultural adaptation commonly involves systematic modification of interventions and training materials to consider language, culture, and context, with the goal of ensuring that it is compatible with the client’s cultural patterns, meanings, and values (23). In order to maintain fidelity to the evidence-based treatment, it is generally recommended that the core intervention components are maintained, while other changes can be made to improve “fit,” in terms of acceptability, comprehensibility, relevance, and completeness (23). In a review of cultural adaptations of treatments for depression, Chowdhary and colleagues (24) found that the most common adaptations were made to language, context, and the person delivering the treatment, rather than to core intervention content.

War Child Holland is conducting an evaluation of a new potentially scalable intervention developed by the WHO, Early Adolescent Skills for Emotions (EASE), in Lebanon. Simultaneously it is being evaluated in three other sites: Tanzania, Pakistan, and Jordan. In Lebanon, the evaluation is part of the STRENGTHS project, that evaluates community-based mental health care implementation strategies to address the psychological needs of Syrian refugees in eight countries (25).

In this paper, we outline the process and results of formative research undertaken to culturally and contextually adapt the EASE intervention for use in the north of Lebanon. By sharing the lessons learned, we aim to contribute to a greater understanding of necessary considerations when adapting psychosocial interventions for use in new settings.

## Methods

### Setting

Lebanon, a middle-income country, has historically experienced significant conflicts, including a civil war from 1975 to 1990, the Hezbollah–Israel war in 2006, and internal conflicts. Additionally, Lebanon now hosts the highest number of refugees per capita globally—with an estimated 1.5 million Syrian refugees, plus large numbers of Palestinian refugees, and vulnerable Lebanese, from a total national population of 5.9 million (1, 26). As a result, there are significant challenges of limited basic infrastructure and the ability to meet educational, health, financial, and mental health needs of the entire population (26). It is estimated that over half of the individuals affected by the Syrian crisis are children; and approximately 1.4 million children in Lebanon are currently growing up at risk, with urgent unmet needs for basic services and protection (26). Most recent estimates indicate only 1.26 psychiatrists and 3.42 psychologists per 100,000 population, with only 3% working in the government sector, making mental health care often inaccessible to the most vulnerable (27).

We conducted our cultural adaptation process in the North governorate, mainly in two vulnerable areas—Beb el Tebbeneh and Hay el Tanak. These were selected as representative areas, given the high vulnerability, and a mix of cultural groups (e.g. Syrian, Lebanese, and Palestinian). When considering cultural and contextual adaptations for psychological interventions in Lebanon, it is important to note that there are vast cultural and contextual differences among different regions of Lebanon including wide variations in income, living conditions, type and opportunities of employment, religion, language, and attitudes toward mental health and associated interventions. Therefore, results of this adaptation process cannot be assumed to be nationally representative.

### Intervention

EASE was developed to address internalizing problems (e.g. depression or anxiety symptoms) in 10- to 14-year-olds living in adversity (28). It consists of seven 90-min group-based sessions for adolescents focusing on four key empirically-supported strategies: understanding my feelings (emotion identification), calming my body (diaphragmatic breathing), changing my actions (behavioral activation), and solving my problems (problem solving). Additionally, three adjunctive caregiver sessions (initially developed to be 120 min each) aim to promote positive parenting practices to improve the caregiver–child relationship and enhance confidence when responding to adolescent distress. Intervention materials consist of: i) facilitator manual for delivering the sessions, ii) workbook for each adolescent to complete individual activities and home practice, iii) storybook to illustrate key concepts, iv) posters for adolescent sessions, and v) caregiver hand-outs.

### Process

**Figure 1:**
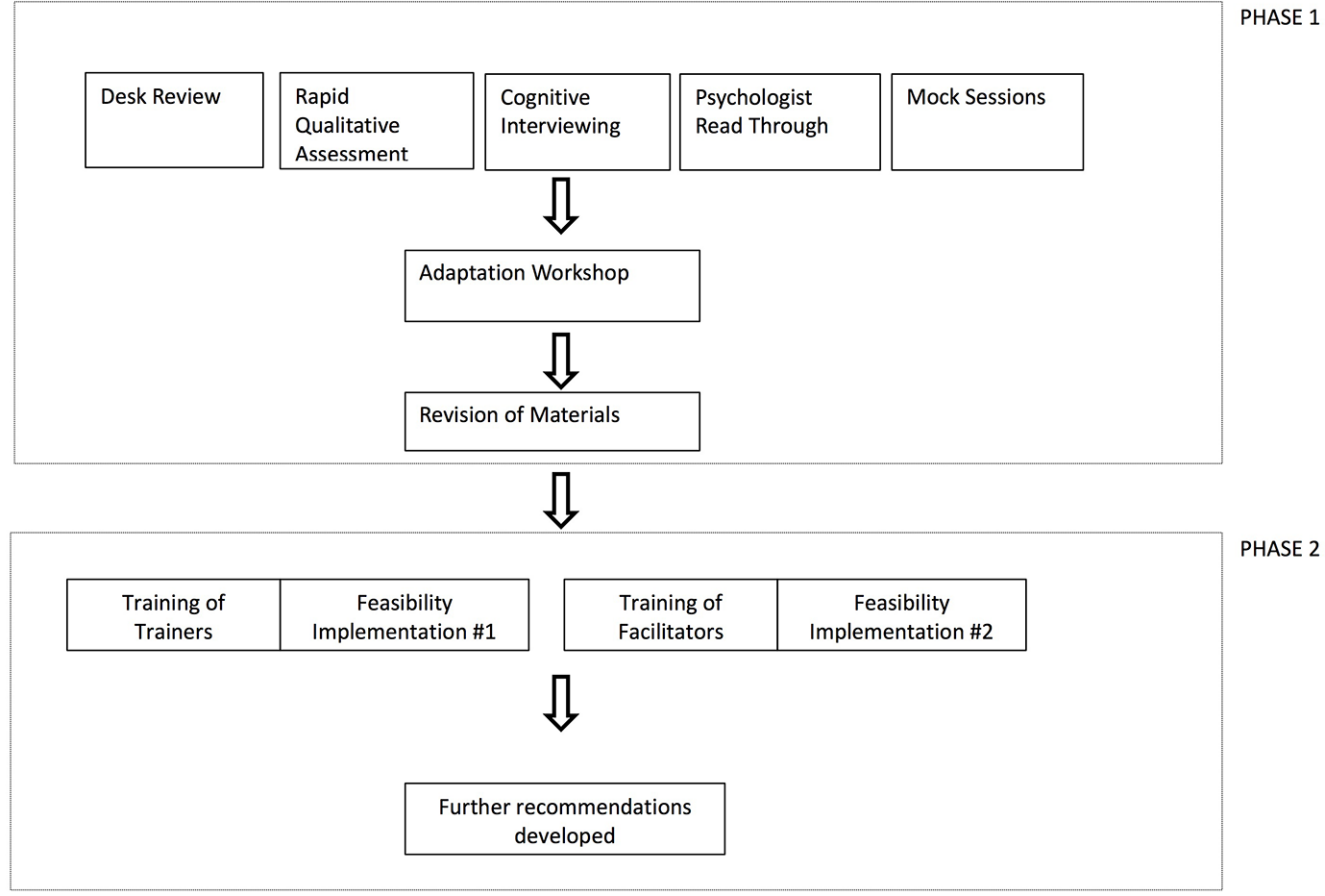
The adaptation process for Early Adolescent Skills for Emotions (EASE) in Lebanon.

We followed an iterative adaptation process based on an internal WHO draft guidance on cultural adaptation of scalable psychological interventions (available on request), which draws from various resources (23, 29, 30). In the first phase, we conducted: i) literature reviews on mental health in Lebanon, with a focus on Syrian refugees; ii) a rapid qualitative assessment (RQA) with adolescents, caregivers, community members, and health professionals in Lebanon; iii) cognitive interviewing with EASE materials; iv) a psychologist Arabic read-through to reach optimal and consistent translation of key terms; and v) “mock sessions” of the intervention with field staff and clinical psychology experts. In an adaptation workshop, data from each of the five steps was reviewed to decide on necessary adaptations. Changes to materials were proposed to developers at WHO, and changes in line with the intervention model were implemented accordingly. The second phase involved: i) gathering data from the Training of Trainers workshop, and associated implementation of practice sessions; and ii) gathering data from the Training of Facilitators workshop, and associated implementation of practice sessions. Several recommendations were developed for further adaptations. This process is depicted in **Figure 1**. Ethical approval was obtained from Saint Joseph’s University, Beirut (USJ-2017-24).

### Phase 1—Formative Research

#### Scoping Review of Literature

In order to learn from existing relevant literature, a two-part non-structured desk review was conducted. One part encompassed reviewing: i) an internal desk review conducted by partners in the STRENGTHS research consortium (available on request), investigating mental health of Syrian refugees in the Middle-East and Europe; ii) existing, known desk reviews pertaining to Syrian child and adolescent mental health (31, 32); and iii) recent internal needs assessments carried out by War Child in Lebanon, not specific to Syrian refugees. The other part took place after the RQA (described below), and further explored common issues identified in the assessment, *via* a non-structured literature search.

#### Rapid Qualitative Assessment

The aims of the RQA were to: i) understand all problems experienced by the community; ii) give insight into how problems related to mental health are expressed locally; iii) explore coping methods commonly used by adolescents, and community coping mechanisms; iv) explore awareness and communication around mental health in the community; v) map available mental health services; and vi) receive input on the planned EASE intervention delivery. The RQA took place in community centers where War Child Holland was implementing education activities.

First, free-listing interviews (29) were conducted with 11 adolescents (aged 10 to 14 years; 8 female, 3 male; 5 Syrian, 6 Lebanese), and 13 caregivers (12 female, 1 male; 4 Syrian, 9 Lebanese), invited through the community center coordinators, who were instructed to find a mixed gender sample that represented the wider community. Trained research assistants asked participants to provide information on the research question “What are all the problems that affect children and adolescents in this community?” with several probes to follow up. Responses were tallied, and the most commonly reported problems that were potentially able to be addressed *via* a psychological intervention, were then explored further through semi-structured interviews with different participants.

Semi-structured interviews were conducted in the format of four focus group discussions with adolescents (age 10–14, *n* = 15; 8 female, 7 male; 11 Syrian, 4 Lebanese); and individual key informant interviews with adolescents (age 10–14, *n* = 7; 3 female, 4 male; 5 Syrian, 4 Lebanese, 1 Palestinian), caregivers (*n* = 9; all female; 4 Syrian, 4 Lebanese, 1 Syrian-Lebanese), community members (*n* = 4; one community center director, one education officer, one football academy leader, and one other respected community member), and health care providers (*n* = 3; clinical psychologist and psychotherapist, psychologist, and social worker). The adolescents, caregiver, and community members were a purposive sample selected by community center coordinators to be either representative of the community (adolescents and caregivers) or knowledgeable about the mental health and psychosocial concerns within the community (community members). The mental health care providers were invited through sector networks in the region. Adolescent participants provided informed assent to participate and their caregivers provided informed written consent. Adult participants gave informed written consent to participate.

Interviews were audio recorded, and interviewers also took detailed notes. Interviews were transcribed verbatim and translated into English *via* a professional translator. At least 10% of transcripts were checked for quality and accuracy by a bilingual team member (KT). The data were analyzed through inductive thematic analysis to determine main themes and illustrative quotes (33). The results are reported in full elsewhere, but pertinent findings are reported here.

#### Cognitive Interviewing

The aim of the cognitive interviewing exercise was to ensure that materials were easily understood, acceptable, and relevant to the population. Two groups of adolescents (*n* = 8) and two groups of caregivers (*n* = 8) were shown the Arabic intervention materials and asked questions about whether they were relevant, understandable, and acceptable, following a semi-structured guide. The materials shown were key intervention components, which were purposefully selected to include components hypothesized by the local team to be less understandable, acceptable, or relevant in Lebanon—talking to young people about suicide, the choice of activity in the story book of “bird watching,” and the explanation of the “Tired Cycle” of behavioral inactivation due to low mood. Data was collated per concept.

#### Psychologist Read-Through

The purpose of the read-through was to ensure consistency, accuracy, and appropriateness of Arabic wording and idioms. The manual was translated to a formal, yet simple, Arabic in order to be understood by Lebanese, Syrians, and Palestinians. Staff from War Child Holland (three psychosocial trainers, the regional psychosocial advisor, and the research coordinator) read through materials and discussed any translation issues together. An existing glossary of mental health idioms and terminology used by Syrians was used as a reference (31).

#### Mock Sessions

Mock sessions were conducted *via* live role-plays, to allow identification of any further necessary adaptations. Attendees included: three psychosocial trainers from Lebanon, the regional psychosocial advisor, three non-Lebanese psychologists, and two non-Lebanese researchers. Each session was role-played, followed by a discussion of comprehensibility, acceptance, completeness, and relevance. This was an addition to the WHO guidance, and recommended based on field experiences indicating that this would provide substantive suggestions for changes (34).

#### Adaptation Workshop

A 2-day adaptation workshop was attended by representatives from organizations involved in the STRENGTHS consortium. The aim of the workshop was to review all data from Phase 1, and determine recommendations for adaptations. Recommendations were compiled for suggested adaptations to the intervention materials, training materials, or implementation considerations, and were coded according to the Bernal framework for cultural adaptation of psychological interventions (23).

### Phase 2—Implementation Data

#### Training of Trainers and Practice Cycle

An 8-day Training of Trainers workshop was held for two trainers from Lebanon (both psychologists). Three master trainers (MA, AM, FB) conducted the training, one of which (MA) is a psychologist from Lebanon.

The two trainers and one master-trainer (MA) each conducted a supervised “practice cycle” to prepare themselves to train and supervise non-specialists in the intervention. This involved delivering the EASE intervention to three groups: one group of females (*n* = 7), one group of males (*n* = 7), and one mixed group (*n* = 5; 3 boys, 2 girls). Adolescents were of mixed nationalities, including Lebanese, Syrian, and Palestinian. Qualitative feedback was gathered from the trainers *via* session notes, supervision notes, and a debrief session at the end of the implementation.

#### Training of Facilitators and Practice Cycle

Two separate trainings were held with facilitators. Training was 9 days, and followed the EASE Training of Facilitators manual. It was followed by a supervised practice cycle implementation, whereby facilitators delivered the intervention to a small number of adolescents. The first training was attended by eight facilitators, with six continuing to the practice cycles. The second training involved nine facilitators, with seven continuing to practice cycles. The non-specialist facilitators did not have any formal mental health qualifications, but all had previous group facilitation experience with children or adults.

During the two supervised practice cycles, 13 facilitators implemented the EASE intervention with a total of 37 adolescents aged 10 to 14 years old, in seven groups (group sizes ranging from 3 to 11 adolescents). Adolescents were not selected specifically for high levels of distress, given that this was the first time the program had been implemented by non-specialists in Lebanon. Facilitators collected attendance data, and completed self-report fidelity checklists of the major components of the sessions, including duration.

Trainers conducted 10 structured observations in total, whereby they rated facilitators on fidelity and competency, using a standardized observation tool developed specifically for EASE (35). Facilitators were rated on whether they completed each component of the session (yes or no), on how well they delivered each section of the session (done well, done partially, needs improvement), and two general competency items which assessed the use of group facilitation skills, and basic helping skills (done well, done partially, needs improvement). Two additional competency items were to be rated if they were applicable—managing an acutely distressed participant, and demonstrating basic safety skills—however these did not arise during any of the observations. Informal qualitative feedback was collected from adolescents, caregivers, and facilitators.

## Results

### Phase 1—Formative Research

#### Scoping Review of Literature

Our review highlighted that conflict-affected Syrians experience a wide range of mental health problems including newly emerged mental health conditions caused by conflict-related experiences, as well as issues related to living conditions (31). Children and adolescents report war-related anxiety, worries about the future, and other emotional distress including grief, frustration and hopelessness (31, 32, 36, 37). Several barriers to accessing health care exist, including language barriers, stigma, and perceived power-dynamics in the therapeutic relationship (31, 32, 36). Children and adolescents experience high rates of family violence, contributing to emotional distress (36). Furthermore, high rates of substance use among adolescents are reported (36, 37). A pervasive daily stressor is poverty, and this is known to affect many other aspects of child and adolescent well-being, including lack of basic needs, poor attendance at school, child labor, substance use, and criminal activity (31, 32, 36, 37). In Lebanon, 70% of Syrian refugees live below the poverty line (26) and reports indicate that poverty and associated experiences can exacerbate hopelessness and frustration, and pre-existing mental health concerns (31, 36). Needs assessments conducted in Lebanon by War Child Holland confirm these findings and highlight the importance of interventions targeting the specific mental health and psychosocial needs of vulnerable populations in Lebanon.

#### Rapid Qualitative Assessment

The most common concerns reported through free listing, that were considered potentially viable to be addressed in a psychological intervention, were: i) physical violence and abuse (among children and adolescents, and from caregivers or teachers toward children and adolescents); ii) neglect and emotional abuse from parents; iii) emotional abuse among children and adolescents (primarily bullying and teasing); and iv) substance use (alcohol, tobacco, and other drugs) among children and adolescents. The framework analysis resulted in seven themes, which are presented in **Table 1**.

**Table 1:** Findings From the Rapid Qualitative Assessment in Lebanon.

Theme	Key findings	Quote
1. Emotional abuse, bullying, and physical violence	Emotional and physical violence were reported by adolescents and adults as very common in the communities. Sexual abuse was also mentioned. Sources of violence could be from teachers at school, within the home, or among children. It was attributed to poor parenting, poverty, and conflict between ethnic groups.	*Of course, emotional abuse. There is no tenderness from the mother, there is beating from the father, there is domination from the brother … What would all this do to the child?* (Community member, female, Lebanese)
2. Substance use	Substance use among adolescents was reported as common, including drinking alcohol, smoking cigarettes, and taking pills. There was limited understanding about the physical and psychological impact of substance use. Perceived causes were boredom, lack of opportunities, poverty, and parenting influences. Perceived effects were an increase in violence, and isolation from family members.	*I mean there is smoking. You feel like there is smoking. You find many people are … I mean I know a kid who is now 14 years old and he smokes, and he has been smoking for about 5 years. I mean it’s not recent*. (Mother, Syrian) *One of its causes is that there are young people who did not continue their studies, they dropped out of school and they have no job, they live in an area where there are no jobs, and they are not studying … one of its results is that they have no money, they become jobless, they drink and do drugs, they behave badly, not to mention that they might also steal*. (Community member, female, Lebanese)
3. Poverty	This was perceived to be a major daily stressor. Poverty and lack of education are cyclical: children drop out of school to work and provide for the family. Families unable to provide essentials such as housing, clothing, food etc. Perceived impact was wide-reaching, with connections mentioned between poverty and abuse, criminal activity, substance use, and emotional problems.	*Yes, it’s true. We have children in school who are no longer in school because their father is not present, and they can’t, so their mother stopped sending them to school, boys in our class, and now they work*. (FGD girls, Syrian)
4. Other problems reported	Tensions between Lebanese and Syrians were reported as a common problem. While Syrians feel discrimination from Lebanese, the Lebanese respondents are dissatisfied with with the Syrian “newcomers.” Stigmatization was reported, and was experienced based on ethnicity, poverty, and mental health problems, Interestingly, there was little mention of traumatic events stemming from the Syrian conflict—though separation from loved ones and disruption of social support were mentioned.	*… I am telling you I now have a complex from the word “That Syrian” and “Those Syrians,” as if “those” are stupid animals, and you don’t feel like a Syrian. We don’t feel like it is a nationality anymore*. (Mother, Syrian)
5. Community awareness of mental health and coping mechanisms	Negative coping mechanisms were reportedly: substance use, violence, and normalization of abuse. Positive coping mechanisms were reportedly: talking with friends and parents (especially mothers), seeking assistance, role of good parenting, taking part in activities, and supportive friendships. There was a varied level of mental health awareness. Only few respondents linked problems to mental health. There were varying opinions on seeking assistance and on the benefits of mental health interventions, they were seen as taboo.	*They talk to their friends and parents the most, but they don’t talk to a hospital, and there are no such centers here* (Boy, Lebanese)
6. Mental health service mapping	Mental health professionals have good knowledge on what is available, but the majority of community members are not aware of the services available. There was a reported suspicion of the work of non-governmental organizations, in terms of quality and fairness to both Syrians and Lebanese, and some respondents felt that such issues were better dealt with from within the community.	*I mean, they need more than just a session, they need more. I mean, more than just a referral to an association that may or may not help them. There are many lacking services*. (Service provider, female, Lebanese)
7. Input on Early Adolescent Skills for Emotions implementation strategies	The facilitator should come from the region, be well trained, and have a relevant background (e.g. mental health professionals). The role of parents is crucial because participation of the adolescents will depend on the willingness of the parents. Perceived suitable locations for EASE included several community structures such as a house, a park, the stadium, or community centers. In terms of scheduling, almost all participants recommend a time outside school and work hours.	*So, we used to check … we used to ask them about the timing of their school, or if they studied in centres, or if they worked … such things*. (Service provider, female, Lebanese)

#### Cognitive Interviewing

Results of the cognitive interviewing indicated that the content was relevant and comprehensible, and the language was simple and easy to understand. Importantly, several findings informed intervention delivery. Adolescents commonly endorsed that psychosocial problems occurred for friends or neighbors, rather than reporting that these problems occurred in their own life. The local team perceived this to be due to a cultural expectation that events occurring within the family home should not be discussed with others. Furthermore, thorough explanation of the EASE strategies was required in order for adolescents to fully grasp them. For the Tired Cycle in particular, explanation on the linkages between mood and behaviors, and the directional nature of the cycle needed to be expanded.

Caregivers were talkative and liked the opportunity to share experiences. Caregivers were worried that suicide would be discussed with their children, as this was perceived to increase the risk of suicide. When they were asked if it is acceptable to talk about suicide, one caregiver said: “With the parents yes, but with the child, no.” Another caregiver further explained: “No because the child will have this idea in their head and they will want to do it!” This highlighted the need for thorough explanation about the reasons for discussing suicide with young people. Physical punishment was openly discussed by caregivers, and reportedly very common, with many stating that it was necessary because they had no other options. Additionally, caregivers recognized the need for taking care of themselves, but found it hard to implement. This highlighted that discussing these topics would be suitable in the intervention, but with sensitivity.

#### Psychologist Read-Through

The language of the EASE materials was generally perceived to be understandable, and several revisions were made for consistency, accuracy, and simplifying terminology. One example was a change in the word for “emotional problems” in Arabic from “*mashakel 3atifiah*” (i.e. emotional problems) to “*mashakel nafsiah*” (i.e. psychological problems), as the first had connotations of “romantic problems.” Furthermore, the concept of suicidality was introduced *via* a culturally appropriate phrase: “Sometimes people have thoughts that their life is not worth living or they wish they would fall asleep and not wake up.”

#### Mock Sessions

Overall the experience of the mock sessions indicated that the content and ideas were largely relevant, acceptable, and understandable in the Lebanese context. Several suggested edits to the original version were identified, to improve facilitators’ ease of use of the manual and increase participant engagement more generally across all contexts. Furthermore, several cultural adaptations were identified for use in Lebanon.

#### Adaptation Workshop

All recommendations for changes from the previous five steps were summarized and discussed, and the changes implemented are shown in **Table 2**. One substantial change to EASE content was adapting the “Tired Cycle” for behavior activation, which previously focused on the inactivity associated with depression. Given the critical prevalence and impact of aggression and violence in the communities, this was adapted to the “Vicious Cycle,” with examples provided for how behavioral cycles can also work to maintain anxiety and anger, and prevent engagement in personally valued activities.

**Table 2:** Adaptations Made in Phase 1 for Early Adolescent Skills for Emotions (EASE) Materials and Implementation in Lebanon (Coded by Bernal Framework).

Adaptation Principle	Change implemented to EASE	Information/Rationale	Source
**LANGUAGE**			
Translation into local language	Ensured consistency in language used for all key strategies, terms like “adolescents” and “youth,” emotional terms.	Consistency will enhance understanding.	Mock Read
Use of local idioms	Considered table of terms used frequently by Syrian refugees, for discussion with facilitators. Added idiom for suicide “*Itmana nam ma fik* (“I wish I could sleep and not wake up”).	Ensures that materials are accessible and easily understood by participants.	Read
Technical terms replaced by colloquialisms	Changed word “client,” to “youth” or “participant” or “caregiver”. Consistency ensured in term “facilitator”—previously was sometimes “helper” or “leader”. “Homework” was changed to “home activities”. Changed “taming feelings” to “managing feelings” to reduce pathologizing. Rephrased “catch” feelings to “notice” or “be aware” of feelings. Changed reference to doctors/scientists to “we know from experience” or “experience shows”.	These terms were identified as most relatable and appropriate in the context, especially given the stigma around mental health interventions. There is stigma and suspicion around mental health services, and a belief that it is better treated within the community.	Mock Read
**PEOPLE**			
Therapist-patient matching	Need to carefully consider whether it is appropriate to have male facilitators with female groups—will pilot and monitor.	Traditions of gender segregation are common.	RQA
Cultural competency of therapists	Facilitators should have experience working in these communities. Sensitivity is needed around the taboo of talking about the family’s problems outside of the home.	It is important that they are well respected, especially given stigma, and reported distrust of NGOs and health services. Adolescents do not often discuss what happens at home outside the home, and often report that these things happen to “other people”	RQA CI
Therapist–patient relationship	Facilitators need to ensure that the tone of interaction between facilitator and caregivers is interactive and inclusive, rather than directive and lecturing. Session content has been edited accordingly. In order to maintain confidentiality and trust, separate facilitators are needed for adolescent and caregiver sessions.	Caregivers enjoyed the opportunity to speak about their concerns. Due to stigma, need to ensure confidentiality of adolescent sessions and ensure they are comfortable to disclose issues.	Mock CI
**METAPHORS**			
Use of material with cultural relevance	Several changes were made to the story to increase cultural relevance. Character was annoyed by his sister joining with his friends—this was changed as not culturally appropriate. Male character doing household chores was adapted to ensure that this could be sweeping outside/around the house, not just indoors. Examples of bullying, anger, and aggression were included as they were highly relevant. Difficult problem of mother being sick, was removed (was potentially intense and upsetting for children experiencing grief) and replaced with problem of family moving house. Replaced asking doctor for help with neighbour/friend/family member. Asking doctor for help is not culturally relevant. Rule setting exercise asks adolescents not to discuss topics such as war, however this was removed as it is very common.	Some aspects of the story were not culturally appropriate. Some adaptations made to increase sensitivity to experiences in this context.	
Use of idioms/symbols	A story about a rabbit hole was removed as not relatable. A detective story that was used to illustrate problem solving was replaced with an interactive maze activity. Children experience hitting dead-ends, and needing to go back and try a different route. Reference to “bright happy” colors was removed.	Some adolescents may not related to these, some adolescents may not associate only “bright” colors with being happy.	Mock
**CONTENT**			
Incorporation of local practices into treatment	In rule setting exercise, removed reference to adolescents pointing to each other’s mistakes. Caregiver sessions reworded to acknowledge that physical discipline is common, and suggest positive discipline alternatives that caregivers can try. More instructions added on ending the program with a celebratory activity.	Encouraging adolescents to point out others’ mistakes is not appropriate in this context. Physical punishment is common in this context, and it is not advisable to tell parents that it is not acceptable, without also including alternative discipline methods. Celebrations are customary in the context.	Mock CI
Addition of therapy modules to address cultural factors	Training added on how to respond (not intervene) when issues of grief, abuse, or substance use arise (how to respond appropriately, providing facilitators with referral information and capacity to recognize issues). Content in session 1 was reduced to allow children time to get comfortable.	Grief, abuse, and substance use are common. They are not addressed anywhere specifically in the manual. Children are not used to discussing their emotions, and need some time and introductory activities to feel comfortable.	Mock Lit RQA
Addressing stressors	Incorporated anger/externalizing problems and aggression throughout. For example: Tired cycle for behavior activation was previously focused on the inactivity associated with depression. This was adapted to the “vicious cycle” (in Arabic—”vicious spiral”), with examples provided for cycles of anxiety and anger/aggression. More focus was added to doing things that are important to you, rather than inactivity. Calming my body strategy suggested for use with anger. Examples of social problems such as anger/aggression or bullying added for problem solving strategy and story. When discussing understanding feelings, added rationale that adolescents sometimes feel bad inside and act out as a result—for both children and caregivers. Examples for caregivers were amended to include mention of externalizing problems.	Physical violence, anger, aggression, and bullying are common problems. We heard examples of negative coping mechanisms in the RQA, but understanding of mental health concerns is lacking, and there are high levels of stigma.	Mock RQA Lit
**GOALS**			
Client derived goals	For the strategy “changing my actions”—facilitators need to be trained on how to help select appropriate activities in this context and setting and how to follow up with each child to make it suitable. Since adolescents are likely to have many problems, a step of prioritization of problems was added, and adolescents are taught to consider problems that adhere to the “3 S’s”—solvable, small, and specific. More varied examples were included in materials, to support use of the strategy.	Poverty and crowded living spaces are pervasive, and children may already be working, therefore suitable enjoyable or meaningful activities may vary. Children have many problems, and many of them are out of their control.	RQA Mock
Clarifying goals	Varied coping strategies were added when discussing helpful and unhelpful coping. Facilitators should be trained to incorporate relevant strategies for specific group. When caregivers are considering adolescent strengths, they should be encouraged to think more broadly than just strengths related to coping strategies.	Coping strategies may vary in the context. Need to consider and encourage broader coping strategies.	RQA Mock
**METHODS**			
Adaptation of training and supervision methods	Detailed training needed for facilitators, to provide explanation and examples of how to discuss strategies with children. Facilitators will need training on how to handle disclosures of abuse. Facilitators will need more training on parenting strategies, to deliver parenting sessions well and to be able to answer questions. They will also need training on how to manage talkative parents.	Facilitators will be non-specialist and will therefore need more detailed training. Facilitators need core understanding and knowledge of children’s well-being and positive parenting. Abuse is common in the community. Parents were very talkative in formative research, and liked the chance to share experiences.	Mock RQA CI
Client engagement adaptations	For child sessions—more interactive methods were added to ensure engagement and attention (e.g. role plays, drawing), and allow interactive group exercises. Explanations for children and caregivers should be simplified as much as possible, and focus on key points only. If the caregiver groups are mixed gender, facilitators should ensure this is considered when making pairs for activities. Caregiver sessions were edited to be more interactive and draw on participant experience, rather than didactic presentations.	In this context, adolescents often do not have space to play and do not attend school. Externalizing problems are also common. Therefore keeping sessions active will increase engagement. Inclusion of interactive group activities will also reduce the individual focus, and broaden the concepts to cover social interactions which are important in this context. Consideration of gender is needed. The children and caregivers will have low literacy and education, and likely will have trouble engaging with lengthy sessions if they are didactic. Concepts should be presented as simply as possible.	RQA CI Lit Mock
Structural adaptations	Scheduling needs to be around school activities, and prayer times.		
Adaptation to techniques used to deliver treatment	Sessions should end with praise for attendance and acknowledgement of effort. Adaptations made to the story book to increase links between sessions and improve the flow of the story. Revision of homework activities to make them simpler, and to increase acceptability of concept for adolescents and caregivers. Need to mention as additional common problems: lack of privacy, caregiver not understanding the importance. Materials revised to ensure that instructions are very clear for facilitators, and more supporting materials (such as posters, and examples) are included Adolescents provided with visual summary of all strategies they have learned	This should be added to end the sessions positively. The story telling element should be strengthened in the story. The amount of homework builds up from each session, so that at the end the child is doing many different things, which will likely be too much for children in this context.	
**CONTEXT**			
Increase accessibility and ensure feasibility	Edits were made to reducing resources needed during sessions—such as balloons, colored pencils, and costumes. For exercises requiring colored pencils, alternative options were provided, in case children did not have them at home. The quality time strategy was adjusted to suit large families, and crowded spaces—acknowledging that quality time may not be one-on-one, and may only be brief. Examples were added of incorporating quality time into daily activities, and facilitator training was added for facilitators about barriers to quality time. Mixed groups of Syrian and Lebanese children should be encouraged, rather than separate groups, as separating groups may further perpetuate inter-group tensions. Facilitators should be trained to manage any tension that may occur in group. Careful community awareness and sensitization activities should be conducted, to reduce stigma, overcome negative perspectives on NGO services, and ensure accurate expectations. Centre should be accessible, and transportation support should be provided to caregivers and adolescents. Childcare should be provided at caregiver sessions.	Poverty is pervasive and families are often large, and living in crowded spaces. One-on-one quality time for lengthy periods is unlikely to be possible. In the RQA, intergroup tensions were identified, as well as mistrust of NGOs, and belief that problems are best dealt with by community. Families have many children, and experience poverty, therefore childcare is likely to be a barrier for caregiver attendance.	Mock RQA Lit
Ensure acceptability	Sections around discussing suicide with youth were carefully worded to ensure caregivers understand that suicide will not be a topic of discussion in the adolescent sessions. When discussing caregiver self-care, references to maintaining a good diet were edited to specify “food is available.” Section encouraging caregivers to watch for warning signs in children in future, was reworded to reduce pathologizing.	Parents may be worried about us talking to children about suicide, when suicide is raised in parent session. Parents say that it’s acceptable to talk about, but worry that might be danger for children. Suicide is a sensitive topic as it is taboo, therefore there needs to be a good link to starting the discussion on suicide. Need to be sensitive to issues of poverty and food shortages.	Mock CI RQA Lit
**SECURITY**			
Specific adaptation relating to conflict-affected setting	Facilitators must be briefed and in contact with security team of organization at all times, and an incident reporting procedure should be in place.	Vulnerable areas in Lebanon are unpredictable in terms of security concerns.	Mock RQA

Mock, mock sessions; RQA, rapid qualitative assessment; CI, cognitive interviewing; Lit, scoping literature review; Read, read through.

#### Translation of Revised Materials

Professional translators translated the revised materials into English. We intended to translate the storybook into simplified, local language. However, based on feedback from trainers and facilitators, it was translated to simple, yet formal written Arabic, and facilitators could adapt the language during delivery. The glossary of key terms in Arabic for EASE, plus common terms for idioms of distress in Syrian Arabic, were provided to facilitators and they were encouraged to use them in their delivery of sessions as appropriate.

### Phase 2—Implementation Data

#### Training of Trainers and Practice Cycles

Through this implementation, it was determined that separation of groups by gender (as recommended by the EASE manual) is beneficial to promote comfort and openness. Similarly siblings and relatives should be separated, where possible, to prevent reticence when discussing personal details. Further, it was determined that the presence of a male facilitator (alongside a female facilitator) was accepted by female adolescents. Significant behavior problems (for example leaving the classroom, and bullying) were experienced in the male group, such that the group was discontinued and individual support provided to interested adolescents. This supports the RQA findings that externalizing problems are salient in this population, and highlights the importance of training facilitators in additional behavioral management strategies in Lebanon. Specific feedback and recommendations for future adaptations to the intervention and implementation are collated in **Table 3**.

**Table 3:** Feedback gathered from Training of Facilitators and Training of Trainers practice cycles and recommendations for further adaptations to Early Adolescent Skills for Emotions materials and implementation in Lebanon.

Implementation considerations		
Topic	Information	Recommendation for implementation in this setting
Literacy	Caregiver and adolescent low education and low literacy were common challenges. Facilitators often had to offer adaptations as required for literacy challenges (e.g. options of drawing).	While the original manual was developed with literacy in mind, further adaptations could be made to address low literacy and education in both child and caregiver sessions—such as adaptations to exercises to reduce reliance on literacy, and simplification of content.
More interaction and active learning	Adolescents did not have many opportunities for active play outside sessisons, and often had interrupted schooling. Adolescents and facilitators indicated a preference for more active tasks in the adolescent sessions, and adolescents particularly enjoyed activities that included drawing and coloring. Caregivers and facilitators also indicated a preference for more group discussion and active learning opportunities in caregiver sessions rather than didactic low literacy were common challenges presentations. Caregivers appeared to take a passive role in the sessions, rather than actively engaging as the key agents of change in their families.	It will be important to increase varied methods of active and interactive exercises to enhance interest and further active-participation of adolescents and caregivers, such as more opportunities for role-play, drawing, and enhancing engagement in the storybook. In this context, there should be a greater emphasis on using energisers, to maintain engagement.
Addressing suicide	There was some concern when caregivers were introduced to the concept of talking to children about suicide—it was felt that this would introduce a new idea to children and lead them to consider suicide. Further, in this culture, suicide is forbidden, and talking about it at all is a taboo.	This topic should be introduced to caregivers in more depth, prior to the commencement, and during the early stages, of the program. Caregivers should be informed about the rationale for talking to children about suicide, and provided with explanations to dispel myths around suicide. Caregivers should be reassured clearly that the EASE sessions do not involve talking with children about the topic of suicide, besides mentioning that if it arises as a concern for a particular child, it will be followed up for their safety, and that it’s covered in the confidentiality explanation for children.
Confidentiality	It became apparent that there was a misunderstanding about the concept of confidentiality. While children were asked to agree to maintain confidentiality of the details shared by their peers within the sessions, and were informed that facilitators would not discuss any details of what they had shared with their caregivers (except when a safety concern arose), children relayed to parents that they had been instructed not to tell parents anything about the session content. Some parents raised concern about this.	In settings where prior experience with health and mental health services is limited, it is important to explain confidentiality clearly, and ensure adequate understanding from children and caregivers.
Referrals	Families were reporting a multitude of other needs, including health, education, food, shelter, and cash assistance.	In settings where other basic needs are often unmet, a list of options for support should be provided to families at the commencement of the program to facilitate access. For Lebanon, there is an existing list of hotlines that families that can call for information on access to services in various sectors.
Group management and behavior management	Adolescents needed more support to work in pairs or groups; given that they may not have attended any school, or limited school, these skills were not always well developed. At times there were significant behavioral issues within the groups (for example leaving the classroom, and bullying).	In this setting, facilitators should provide further guidance and support for adolescents in pair and group work and not assume that this is a pre-existing skill set. Adequate time should be spent on establishing group rules and expectations. Facilitators should be well trained in behavior management in group settings. Clear guidance is needed regarding situations where a child displays severe challenging behaviors.
Simplification of manual	The format of the manual was challenging to follow. Facilitators recommended that the Arabic translation could be improved *via* simplifying terms to those more commonly used in everyday language.	The manual should be as simple to read and use as possible. Arabic translation could be simplified.
Poor attendance	Low attendance rates were observed. There were challenges where children or caregivers had missed a session and attended a later one, because limited time was available to cover the missed material. Some caregivers reported that transportation to sessions was still challenging even when provided with a reimbursement for costs, as they did not always have money available for their trip.	Participants should be briefly informed of what the main activities were when they missed a session, and time will be needed for this. Appropriate scheduling of group sessions is essential, and considerations must be made for competing activities such as school, prayer, common employment times, and other recreational programs. Strategies need to be in place to remind and encourage both caregivers and adolescents to attend, including phone calls prior to each session. Transportation support should be arranged for caregivers.
Session length	Caregivers reported that 2 h (plus travel time) was too long to be away from home. Facilitators reported that the initial child sessions were too long. Participants were often eager to return home or back to other activities. Facilitators reported that the content was not able to be completed adequately in the time allowed—Sessions were sometimes ended early or were not completed.	It is recommended that briefer sessions, with reduced content, be scheduled in this setting where caregivers have many competing responsibilities.
Comfort around discussion of emotions	There was discomfort around discussing emotions with others, and shyness about discussing issues, especially during earlier sessions. Facilitators requested an introductory session for adolescents.	Rather than adding an additional session (which would increase resources required) it is recommended that adequate time is provided during the first session to allow children to “warm up,” either *via* increased time allotted, or reduction of content.
Homework challenges	Adolescents and parents had trouble completing homework.	The instructions for homework tasks should be as simple as possible, and the importance of adequate review and troubleshooting of homework at the start of each session should be emphasized to facilitators. To facilitate home practice involving identification of emotions, a poster which displays a range of feelings, can be provided to children to take home.
Requests for celebration	Facilitators, trainers, adolescents, and caregivers requested a celebration session at the end of the program, in line with common practice in this setting. Caregivers requested incentives for adolescents to participate. Adolescents were very excited by certificates.	A small celebration could be added to the final session, with a small refreshment, and certificate presentation to mark the completion of the program. To maintain motivation, the certificate could be mentioned to adolescents at the beginning of program.
Group compositions	Separation of groups by gender is beneficial to promote comfort and openness. Siblings and relatives should be separated where it is possible to prevent reticence when discussing personal details. The presence of a male facilitator in the female group was accepted.	It is important to consider group composition in terms of gender matching of participants and facilitators, and family members.
Delivery of specific strategies	Adolescents had difficulty with the following aspects of discrete strategies: identifying strengths, often naming skills instead identifying a range of emotions, and recalling emotions across a full day managing dizzy feelings, and focusing for long periods during breathing exercises personally relating to the vicious cycle breaking specific tasks down into smaller steps for behavioral activation remembering to seek adult support in problem solving strategy	It is important that facilitators are skilled in using prompts and questions to support adolescents and caregivers with common challenges, are able to describe strategies in alternative ways, and able to provide a multitude of examples.

#### Training of Facilitators and Practice Cycles

On average, session duration was between 1.5 and 2 h. Attendance was variable, particularly for later children and caregivers sessions, ranging between an average of 53% and 89% per session. Facilitator-reported fidelity to the major components of the intervention was generally high, with low-fidelity reported for some specific sessions, sometimes due to the shortening of session time due to practical issues, or only one participant attending and therefore some group activities not being completed.

Trainer-observed fidelity was slightly lower, however overall, most facilitators were implementing the majority of the components in most sessions. Some items were not completed due to lack of time. Competency ratings for delivery of EASE content were rated high, and the majority of ratings on the core competencies of group facilitation and basic helping skills were the highest possible score.

Facilitators gathered and documented feedback from adolescents and caregivers at the end of the program. Overall, feedback was positive. Adolescents found the problem-solving strategy (managing my problems) most useful, and were commonly practicing diaphragmatic breathing. Some adolescents could not personally relate to the vicious cycle, which described the link between mood and detrimental behavior patterns that took them away from meaningful activities. In cases where children could not identify meaningful activities that were being impacted, facilitators asked children to use the behavioral activation strategy (changing my actions) to increase pleasurable activities, or improve everyday planning. Caregivers reported finding the diaphragmatic breathing strategy, and the exercise about caregiver strengths useful and felt that they were using more supportive and less harsh parenting techniques. Caregivers reported challenges around the implementation of quality time, given the large family sizes and time limitations and facilitators handled this as specified in the manual in terms of emphasizing brief moments. Specific feedback and recommendations for future adaptation to be implemented by WHO, are collated in **Table 3**.

## Discussion

The aim of this study was to culturally and contextually adapt the EASE program for young adolescents in the north of Lebanon. We conducted a scoping review of the literature, RQA, cognitive interviewing, psychologist read-through, and mock sessions, and gathered data and feedback during training and practice cycles. Based on the findings, we made several minor and several more substantial adaptations to the materials and implementation methods.

Our study lends support to the utility of conducting a careful and rigorous adaptation process before delivering an intervention in a new setting. We identified several important adaptations to key components of EASE that enhanced relevance and completeness in addressing the common experiences of adolescents in this community. First, the most substantial change to content of adolescent EASE sessions was the incorporation of examples of anger and bullying throughout the materials, given the high prevalence and impact. This notably included adapting the inactivity cycle of depression, to the broader “vicious cycle” which incorporates processes maintaining a spectrum of emotional distress behaviors (including anger and anxiety). Behavior activation is considered to be an effective and cost-effective technique suitable for wide-scale dissemination by non-specialists (38). However, while the strategy is commonly used to address inactivity associated with depressed mood, it has been previously noted that expanding the focus away from depressed mood and toward encompassing a broader concept of maladaptive behavior patterns interfering with valued activities, may reduce the impact of stigma related to depression, and increase cross-cultural applicability and relevance (39). This is in line with evidence that presentations of depression symptoms vary in different cultural contexts, and also in populations exposed to trauma (40). It also corresponds to adaptations made to an online psychological intervention for adults in Lebanon, where the focus was shifted away from inactivity as a symptom of depressed mood, with emphasis instead on increasing pleasurable activities to lift mood (41). Second, given the number and intensity of problems faced by adolescents in this context, with many outside the adolescents’ personal control (e.g. poverty, community violence), facilitators were required to more actively support adolescents to identify and prioritize appropriate problems for the problem-solving strategy. Third, the most significant change to the caregiver EASE content was the consideration of large families and limited physical space for the quality time strategy. Facilitators encouraged parents to keep quality time brief, and to accommodate for times where other siblings will also be present, by ensuring that individual attention is provided to each child. Fourth, given parents’ low literacy and education background, the amount of session content was reduced in order to retain only the key strategies and concepts.

Several changes were also recommended for session delivery. Given that adolescents often had a lack of opportunity for active play, and often had interruptions to schooling which meant that they were not accustomed to attending for long periods, there was a need for activities to be more physically active and interactive in order to maintain focus and engagement. Specific guidance should be given for facilitators on setting up group rules, and coaching children in group and pair work. Similarly for caregivers, it was recommended to increase interaction and reduce didactic presentations.

The types of adaptations made for EASE, are similar to adaptations made in other cultural adaptations in the mental health field. A recent systematic review of depression interventions for adults found that the most commonly adapted components were related to: language, facilitator, and context (24). EASE content was already simplified with non-specialists in mind, however gender matching of facilitators was carefully considered. Additional training for non-specialist providers was developed, covering topics of behavior management, and responding to disclosure of grief, substance use, and abuse. Significant adaptations were made based on context, including considerations around requirement of resources such as colored pencils, balloons, and costumes.

Based on our experiences, we provide several key recommendations for the process of cultural and contextual adaptation of psychological interventions. Firstly, when developing psychological interventions for use across contexts, material should consider low literacy and education levels of participants, low resources available, and non-specialist training of providers. Additionally, while materials will never be devoid of culture, several features can be incorporated to enable easier adaptation in new contexts, including: i) explicit mention of origin of materials including evidence for the use of each strategy; ii) specification of discrete sections that will likely need cultural adaptation (e.g. case stories that can be easily replaced); or iii) developing materials such that they are relevant in several different cultural contexts (e.g. key characters and illustrations used in the material are gender-neutral, age-ambivalent, without culturally important details that are not significant for the message that is being conveyed). Secondly, the mock sessions gleaned numerous useful suggestions for cultural adaptation as well as improvement of materials more broadly, and therefore are recommended as an important and cost-effective step, prior to training of facilitators and translation of final products.

We experienced several significant challenges during the adaptation process. Foremost, we had challenges relating to using formal written Arabic compared to colloquial versions. Initially we intended to translate materials into simplified and more local language, however facilitators reported a unanimous preference to receive the materials in simple, formal written Arabic, and would adapt the language during delivery to suit participants. Some facilitators were Lebanese and others were Syrian, while many of the adolescents in the program were Syrian. Therefore, exact matching of accent and colloquial terms used between facilitator and adolescents was not achievable. It is important to further note that Arabic dialects vary by region and by socio-economic status in Lebanon. Future work in Lebanon should aim to engage bilingual individuals with a strong mental health background as well as extensive knowledge of local languages, in order to optimally adapt terminology to be understood by facilitators, adolescents, and caregivers in these diverse communities.

One of our major challenges, relating to uptake and attendance at sessions, was scheduling implementation of EASE sessions around school and work commitments. The Ministry of Education and Higher Education in Lebanon has implemented double-shift schools in Lebanon, in a response to vastly increased numbers of school-age children and adolescents following the Syrian crisis, meaning that children were attending school at different times of the day. Additionally, high rates of child labor presented a significant challenge for attendance. Unfortunately, literature indicates that child labor is a risk factor for increased psychological distress and other protection needs (3). Our experience supports a need for integrated multi-sector programming that includes efforts to reduce child labor within communities, as well as providing multiple options for mental health services, to ensure that the most vulnerable children and adolescents are being reached. This may include flexible one-to-one sessions for those that cannot attend group sessions, or online or telephone options. A group format was selected for EASE in order to reduce resources needed for delivery and increase coverage, however future implementation research should evaluate cost-effectiveness, taking into account attendance rates in different settings, and the likely consequent attenuated impact associated with poorer attendance.

Our experience during the adaptation process conducted for EASE elucidates a core dilemma in delivering culturally sensitive treatments. On the one hand, there are significant cost-benefits and quality assurance advantages to maintaining core base materials for scalable interventions, which can be readily adapted to new contexts and cultures, and this may be a pre-requisite for replication of evidence-based treatments. Furthermore, developing consistent core intervention materials for use across various contexts, more readily enables development of global training and supervision networks, and effective integration of improvements and lessons learned from different settings. On the other hand, Kirmayer (17) argues that the evidence underlying evidence-based treatments is grounded in particular cultural assumptions that are deeply embedded in our diagnostic frameworks, interventions, and measured outcomes, and therefore does not adequately address cultural diversity. From this view, a more ground-up approach may be preferable for optimally incorporating cultural understandings and processes of illness and healing into our practice (17, 42). Rousseau and Kirmayer (42) caution against attributing the efficacy of culturally adapted treatments solely to the underlying psychotherapeutic framework, and instead encourage evaluation of the added cultural elements as explicit active ingredients for change themselves.

The EASE intervention was developed primarily for internalizing symptoms of depression and anxiety, and therefore during our adaptation process there was limited scope to add alternative strategies or significant focus on the problems reported during our formative work in vulnerable communities in Lebanon (e.g. significant prevalence of externalizing symptoms). Conversely, there is significant comorbidity between externalizing and internalizing problems, and increasing evidence for transdiagnostic approaches to address both (43). In the Lebanon context, where externalizing problems may be prominent, it will be important to understand the impact of the transdiagnostic components in EASE on both externalizing and internalizing symptoms. An additional key recommendation for future implementation and research is that early formative work in a new setting (including literature reviews and RQAs) should be conducted prior to selecting or developing new interventions to adapt and apply, based on identified context-specific priorities and understandings. It is imperative that flexible funding structures allow for such careful cultural enquiry prior to the selection of evidence-based approaches to implement.

Overall, our experience with adaptation of the EASE program in the north of Lebanon is promising and indicates the suitability of a brief, non-specialist delivered intervention for adolescents and caregivers. The research informs the wider field of global mental health in terms of opportunities and challenges of implementing psychological interventions in diverse settings of low resources and high adversity, and the potential to contribute to closing the global treatment gap (4). Child and adolescent mental health continues to be an area lacking sufficient attention, especially in under-resourced settings (7), and the results of this formative work, and forthcoming pilot trial and randomized controlled trial will provide further evidence on effective strategies to address this global issue (35).

## Data Availability Statement

The datasets generated for this study are available on request to the corresponding author.

## Ethics Statement

The studies involving human participants were reviewed and approved. Ethical approval was obtained from Saint Joseph’s University, Beirut (USJ-2017-24). Written informed consent to participate in this study was provided by the participants’ legal guardian/next of kin.

## Author Contributions

The overall process for the adaptation of EASE was designed by FB and MJ, with contributions from all authors. FS, MB, and PH contributed to the Desk Review. The Rapid Qualitative Assessment was overseen by FB and MJ, with FB and FS responsible for training research assistants, FS and KT responsible for data collection, FS contributing to data analysis alongside a master’s student, and field support provided by MA. The cognitive interviewing was overseen by FB and MJ, with KT responsible for data collection and analysis, and support provided by MA and FS. The psychologist read-through was coordinated by KT, with support from MA, and other staff members. Mock sessions were coordinated by FB; were conducted by FB, MA, AM, and PH; and attended by FS, KT, and MB. The adaptation workshop was coordinated by FB, and attended by MA, AM, PH, MB, MJ, FS, KT, and RC. Several authors (AM, KD, SW, FB, MA, PH) contributed to edits to materials, with WHO technical staff in Geneva providing the final sign-off. Training of Trainers (TOT) was conducted by MA, FB, and AM. MA and two other trainers implemented TOT practice cycles and shared valuable feedback. TOF was conducted by MA, and two trainers. MA and FB were responsible for supervision and collecting all field data and recommendations from the TOF practice cycle, with support from trainers and KT. MA, FB, and AM provided “master supervision” and collected feedback throughout. MS led the development of the overall STRENGTHS consortium plan, including cultural adaptation components. All authors critically reviewed and approved the manuscript for publication.

## Funding

This project has received funding from the European Union's Horizon 2020 research and innovation programme under grant agreement No 73337.

## Conflict of Interest

The authors declare that the research was conducted in the absence of any commercial or financial relationships that could be construed as a potential conflict of interest.
